# Selective depletion of microglial progranulin in mice is not sufficient to cause neuronal ceroid lipofuscinosis or neuroinflammation

**DOI:** 10.1186/s12974-017-1000-9

**Published:** 2017-11-17

**Authors:** Terri L. Petkau, Natalia Kosior, Kathleen de Asis, Colúm Connolly, Blair R. Leavitt

**Affiliations:** 1 Centre for Molecular Medicine and Therapeutics, Department of Medical Genetics, University of British Columbia, and Children’s and Women’s Hospital, 980 West 28th Avenue, Vancouver, BC V5Z 4H4 Canada; 2 Division of Neurology, Department of Medicine, University of British Columbia Hospital, S 192 - 2211 Wesbrook Mall, Vancouver, BC V6T 2B5 Canada; 3 Brain Research Centre, University of British Columbia, Vancouver, BC V6T 1Z3 Canada

**Keywords:** Frontotemporal lobar degeneration, Neuronal ceroid lipofuscinosis, Progranulin, Conditional knockout mice, Neuropathology, Lysozyme promotor, Microglia

## Abstract

**Background:**

Progranulin deficiency due to heterozygous null mutations in the *GRN* gene are a common cause of familial frontotemporal lobar degeneration (FTLD), while homozygous loss-of-function *GRN* mutations are thought to be a rare cause of neuronal ceroid lipofuscinosis (NCL). Aged progranulin-knockout (Grn-null) mice display highly exaggerated lipofuscinosis, microgliosis, and astrogliosis, as well as mild cell loss in specific brain regions. In the brain, progranulin is predominantly expressed in neurons and microglia, and previously, we demonstrated that neuronal-specific depletion of progranulin does not recapitulate the neuropathological phenotype of Grn-null mice. In this study, we evaluated whether selective depletion of progranulin expression in myeloid-lineage cells, including microglia, causes NCL-like neuropathology or neuroinflammation in mice.

**Methods:**

We generated mice with progranulin depleted in myeloid-lineage cells by crossing mice homozygous for a floxed progranulin allele to mice expressing Cre recombinase under control of the LyzM promotor (Lyz-cKO).

**Results:**

Progranulin expression was reduced by approximately 50–70% in isolated microglia compared to WT levels. Lyz-cKO mice aged to 12 months did not display any increase in lipofuscin deposition, microgliosis, or astrogliosis in the four brain regions examined, though increases were observed for many of these measures in Grn-null animals. To evaluate the functional effect of reduced progranulin expression in isolated microglia, primary cultures were stimulated with controlled standard endotoxin and cytokine release was measured. While Grn-null microglia display a hyper-inflammatory phenotype, Lyz-cKO and WT microglia secreted similar levels of inflammatory cytokines.

**Conclusion:**

We conclude that progranulin expression from either microglia or neurons is sufficient to prevent the development of NCL-like neuropathology in mice. Furthermore, microglia that are deficient for progranulin expression but isolated from a progranulin-rich environment have a normal inflammatory profile. Our results suggest that progranulin acts, at least partly, in a non-cell autonomous manner in the brain.

**Electronic supplementary material:**

The online version of this article (10.1186/s12974-017-1000-9) contains supplementary material, which is available to authorized users.

## Background

Loss-of-function mutations in the progranulin (*GRN*) gene cause neurological disease in patients, typically frontotemporal lobar degeneration (FTLD) for heterozygous-null mutations [1, 2], and neuronal ceroid lipofuscinosis (NCL) in the rare case of homozygous-null mutations [3]. Neuropathological analysis of patients with *GRN*-dependent FTLD reveals neuronal cell loss primarily affecting the frontal and temporal lobes of the brain, increased microgliosis in affected brain regions, and TDP-43 pathology [4]. Typical neuropathological features in NCL include early and robust microgliosis, astrogliosis, and lipofuscinosis in the thalamus, which spreads to other brain regions and is ultimately followed by extensive neuronal cell loss [5]. NCL neuropathology is not yet confirmed in *GRN*-null patients [3] but inferred based on the consistency of clinicopathological features shared by *GRN* mutation carriers and patients with other genetic causes of NCL and the consistency of neuropathological features seen in other forms of NCL, their respective mouse models, and Grn-null mice (reviewed in [6]).

Progranulin, a secreted glycoprotein with ubiquitous expression and pleiotropic actions in the body [7], is expressed in most neuronal populations and in microglia in the brain [8] and plays a role in lysosome biology [9]. Mice constitutively null for the homologous murine progranulin gene (*Grn*) display robust neuropathological features consistent with NCL, including microgliosis, astrocytosis, and exaggerated deposition of NCL-like autofluorescent pigment and/or lipofuscin, occurring earliest and most notably in the thalamus and later becoming widespread throughout the brain [10–15]. Behavioral changes in Grn-null mice are modest and somewhat inconsistent, though social dominance deficits [16–18] and obsessive compulsive-like (OCD-like) behaviors are consistently observed [18].

The relative contributions of neuron-derived and microglia-derived progranulin to behavioral and neuropathological phenotypes are a recently emerging area of investigation. A deficit in social dominance is the only reported behavioral change in heterozygous Grn-null mice [17], a phenotype that is recapitulated in neuron-specific Grn knockout mice [16]. Notably, the neuropathological features that robustly define Grn-null mice are not observed in heterozygous Grn-null nor neuronal-specific Grn knockout mice [16, 19].

With respect to microglia, increased self-grooming, an OCD-like behavior that is partially regulated by the inflammatory cytokine TNFα, was recapitulated in mice with progranulin knocked down specifically in microglia [18]. Importantly, this study also observed deficits in social behavior that were not present in microglia-specific Grn-knockout mice, reaffirming that behavioral phenotypes can arise due to progranulin deficiency in a single cell type.

In this study, we use mice with selective depletion of progranulin in myeloid-lineage cells, including microglia, to evaluate the NCL-like neuropathological phenotypes observed in constitutive Grn-null mice. We find no evidence of neuropathological changes in myeloid-specific Grn-targeted mice despite robust neuropathology in Grn-null mice. The overall reduction of progranulin expression in the brain in myeloid-specific Grn-targeted mice was moderate, which led us to examine gene expression changes specific to microgliosis and lysosomal dysfunction, as well as the inflammatory phenotype of primary microglia cultures in response to a stimulus, in myeloid-specific Grn-targeted mice. In all cases, we measured robust changes in Grn-null mice but not in myeloid-specific Grn-targeted mice. We provide evidence that some progranulin-dependent phenotypes are non-cell autonomous, adding an additional level of complexity to progranulin biology in the brain.

## Results

### Progranulin expression is decreased in Lyz-cKO mouse microglia

**Fig. 1:**
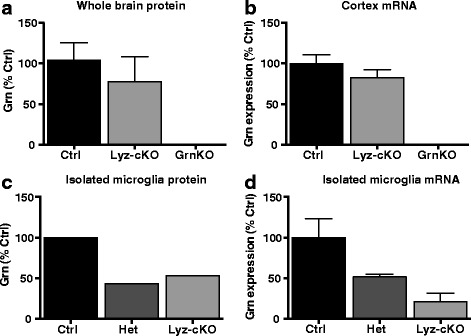
Grn expression is significantly reduced in microglia in Lyz-cKO mice. **a** Grn measured in whole brain lysate by ELISA. *N* = 10 Ctrl, 6 Lyz-cKO, and 4 GrnKO mice. **b** Grn mRNA measured by qRT-PCR in cortex RNA samples. *N* = 4 Ctrl, 3 Lyz-cKO, and 2 GrnKO mice. **c** Grn measured by ELISA in adult microglia isolated by flow cytometry. Microglia from 4 mice per genotype were pooled and run as a single sample in duplicate. **d** Grn mRNA measured by qRT-PCR in RNA extracted from adult microglia isolated by flow cytometry. *N* = 4 Ctrl, 4 Het, and 4 Lyz-cKO mice. Data represent mean ± SEM

Progranulin is primarily expressed in both neurons and microglia in the brain [8]. Although cellular levels of microglial progranulin expression are relatively higher than neuronal expression levels [8, 20], microglia account for a much smaller proportion of cells in the brain. We measured overall brain levels of *Grn* mRNA and protein and found that progranulin is reduced by approximately 20–30% in Lyz-cKO mice (Fig. 1a, b). This moderate reduction in overall levels was expected; however, to verify more robust knockdown in the cell type of interest, we isolated microglia from the brains of 3-month-old mice by flow cytometry and again measured *Grn* mRNA and protein levels. In isolated microglia, progranulin expression was reduced by approximately 70% compared to Ctrl at the mRNA level and approximately 50% of Ctrl at the protein level (Fig. 1c, d).

### Neuropathology in Lyz-cKO mice does not replicate that of GrnKO mice

**Fig. 2:**
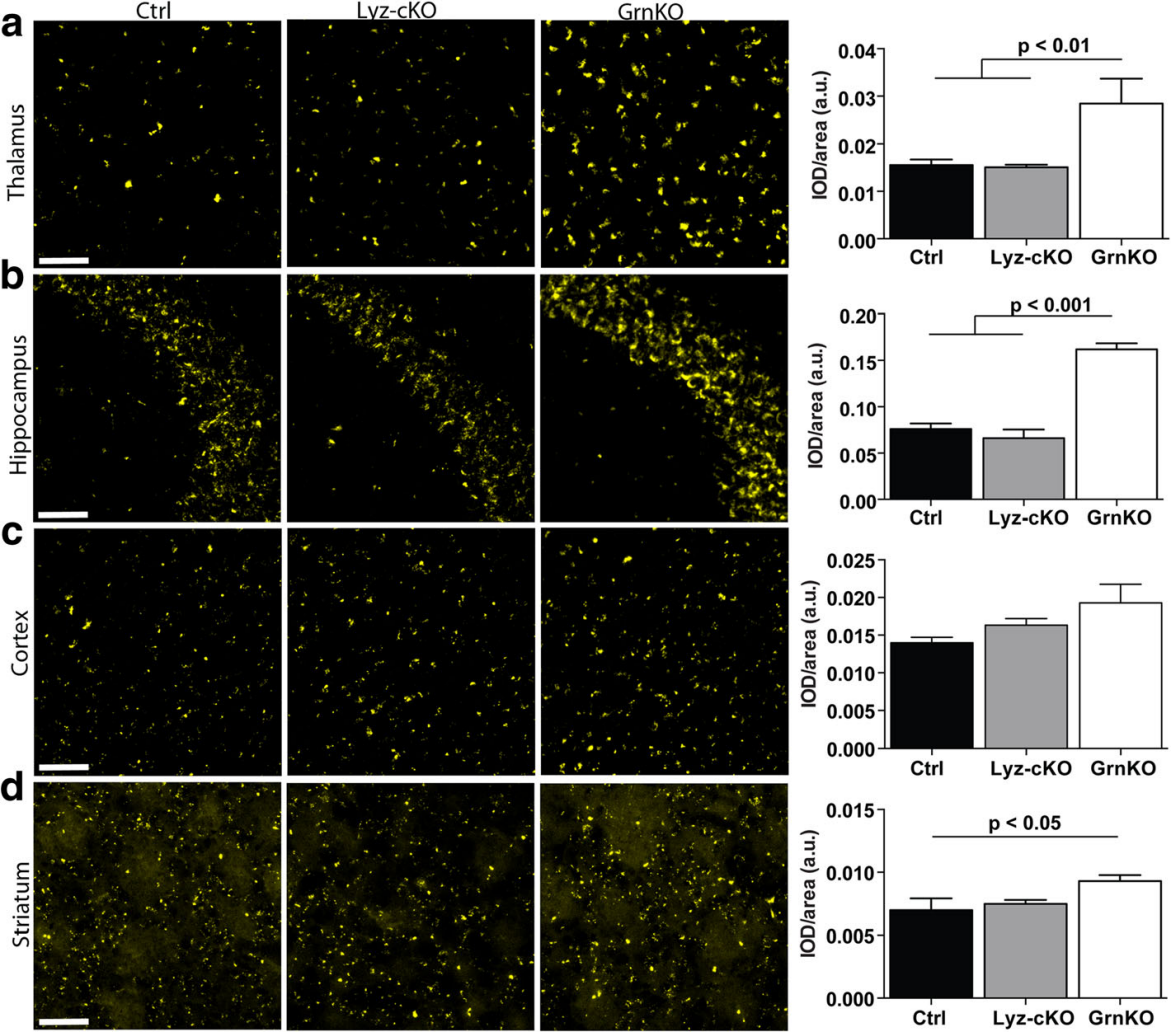
Exaggerated lipofuscin deposition in GrnKO mice is not recapitulated in Lyz-cKO mice. Representative images of autofluorescence, a surrogate for lipofuscin/NCL-like storage material, in the brains of 12-month-old WT, Lyz-cKO, and GrnKO mice are shown for the thalamus (**a**), CA3 region of the hippocampus (**b**), cortex (**c**), and striatum (**d**). In each case, quantification of the images is given in the right-most panel. Data is presented as the average integrated optical density (IOD) over the area measured in arbitrary units (a.u.). For each mouse, 4–6 images per region were measured and averaged to give a single value per animal per region. *N* = 5 Ctrl, 12 Lyz-cKO, and 4 GrnKO mice. Data represent mean ± SEM. *p* values were calculated using Tukey’s multiple comparison test after one-way ANOVA

Complete loss of progranulin expression in the brain causes neuropathology in mice that mimics that of NCL; namely, exaggerated deposition of autofluorescent storage material and lipofuscin, as well as increased microgliosis and astrogliosis, which occur throughout the brain but are most prominent in the thalamus [12]. We quantified the amount of autofluorescence in four different brain regions as a surrogate for NCL-like storage material/lipofuscin accumulation. Autofluorescent material is detectable in the thalamus of Ctrl animals and is significantly increased in GrnKO animals (Fig. 2a). In the thalamus of Lyz-cKO mice, the level of autofluorescence was comparable to that of Ctrl mice. Similar results were seen in the CA3 region of the hippocampus (Fig. 2b) and striatum (Fig. 2d). In the cortex, the level of autofluorescence showed a similar trend, though the increase in GrnKO did not reach statistical significance (Fig. 2c).

**Fig. 3:**
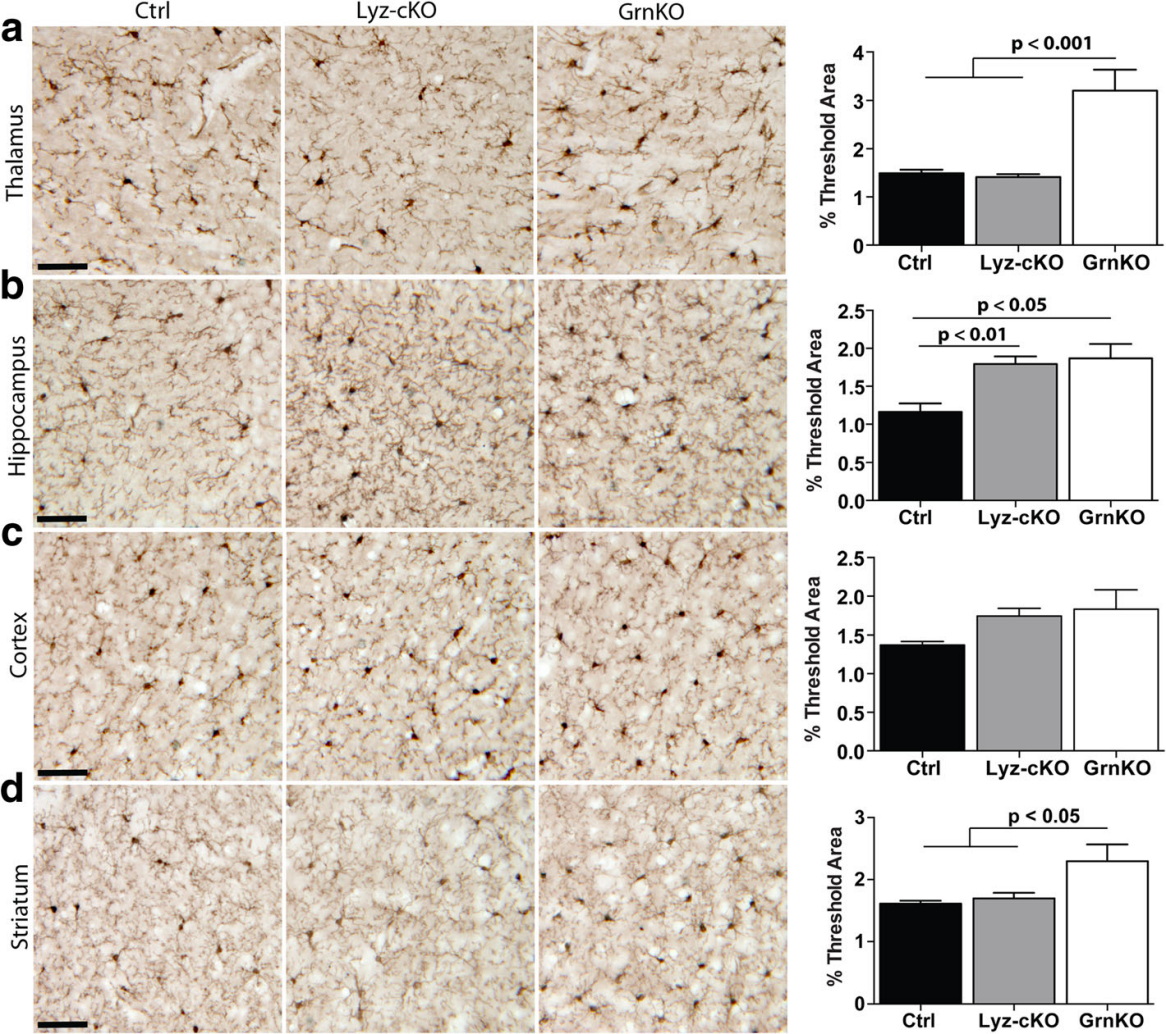
Increased microgliosis present in GrnKO mice is not present in Lyz-cKO mice. Representative images of Iba1 immunoreactivity in the brains of 12-month-old WT, Lyz-cKO, and GrnKO mice are shown for the thalamus (**a**), CA3 region of the hippocampus (**b**), cortex (**c**), and striatum (**d**). In each case, quantification of the images is given in the right-most panel. For each mouse, 4–6 images per region were measured and averaged to give a single value per animal per region. *N* = 5 Ctrl, 12 Lyz-cKO, and 4 GrnKO mice. Data represent mean ± SEM. *p* values were calculated using Tukey’s multiple comparison test after one-way ANOVA

Microgliosis was assessed by quantifying Iba1 immunoreactivity in the same four brain regions. As expected, the thalamus showed a significant increase in Iba1 immunoreactivity in GrnKO mice compared to Ctrl controls, but the level of Iba1 staining in Lyz-cKO animals was similar to that of Ctrl mice (Fig. 3a). In the CA3 region of the hippocampus, Iba1 immunoreactivity was increased relative to Ctrl in both Lyz-cKO and GrnKO mice (Fig. 3b). In the cortex, no significant differences in Iba1 staining were observed (Fig. 3c), while in the striatum, we observed a significant increase in Iba1 staining in GrnKO animals but not in Lyz-cKO animals compared to Ctrl mice (Fig. 3d).

**Fig. 4:**
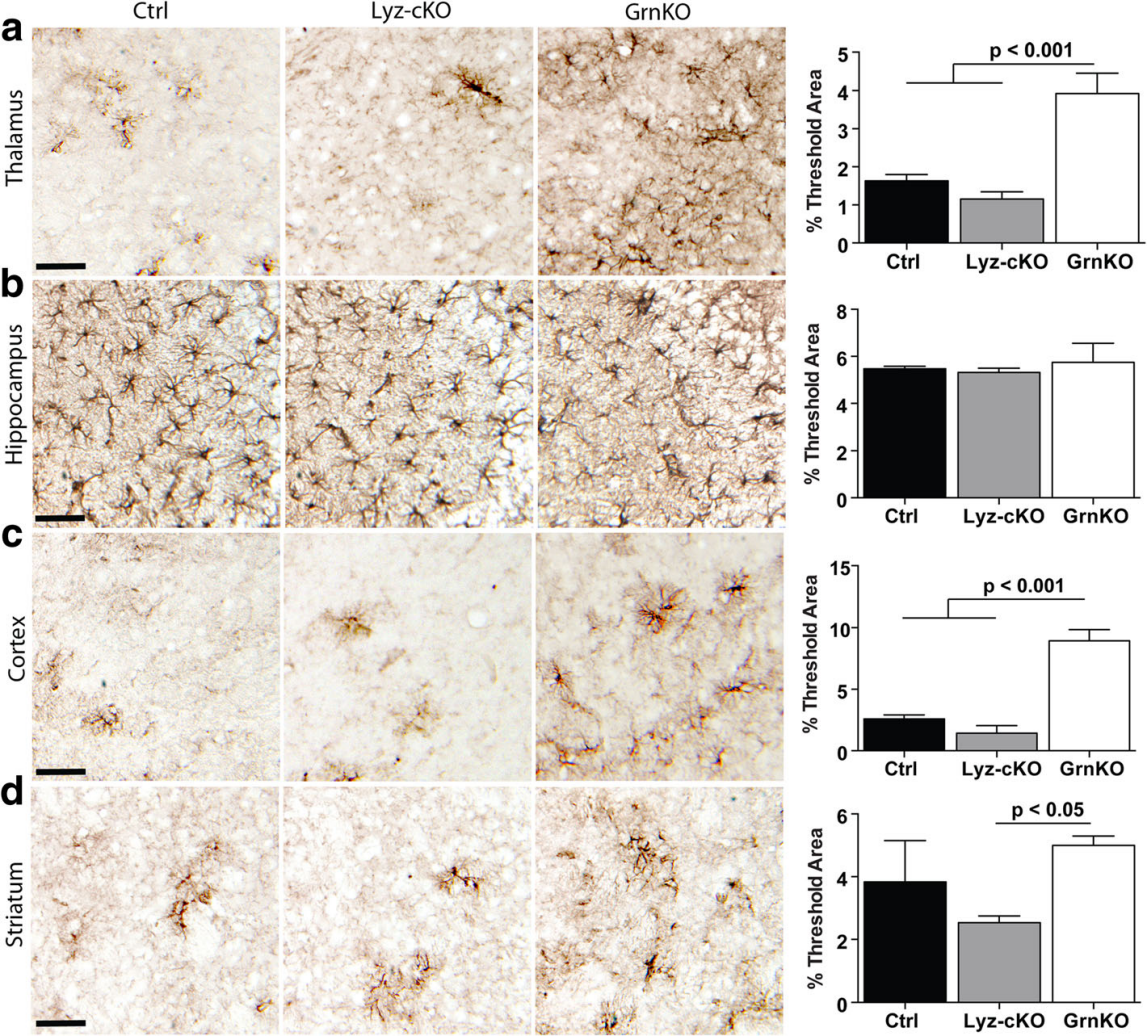
Increased astrogliosis present in GrnKO mice is not present in Lyz-cKO mice. Representative images of GFAP immunoreactivity in the brains of 12-month-old WT, Lyz-cKO, and GrnKO mice are shown for the thalamus (**a**), CA3 region of the hippocampus (**b**), cortex (**c**), and striatum (**d**). In each case, quantification of the images is given in the right-most panel. For each mouse, 4–6 images per region were measured and averaged to give a single value per animal per region. *N* = 5 Ctrl, 12 Lyz-cKO, and 4 GrnKO mice. Data represent mean ± SEM. *p* values were calculated using Tukey’s multiple comparison test after one-way ANOVA

Finally, we quantified GFAP immunoreactivity to evaluate astrocytosis in the brain. In both the thalamus (Fig. 4a) and cortex (Fig. 4c), GFAP immunoreactivity was significantly increased in GrnKO mice compared to Ctrl, while in Lyz-cKO mice, there was no increase compared to Ctrl mice. No difference in GFAP staining was observed between the three genotypes in the CA3 region of the hippocampus (Fig. 4b). In the striatum, there was significantly increased GFAP staining in GrnKO mice compared to Lyz-cKO mice (Fig. 4d), though the increase was not significantly different from Ctrl mice, where the quantity of GFAP staining was highly variable.

Overall, the neuropathological phenotype of GrnKO mice is consistent and robust, with increased deposition of lipofuscin/NCL-like storage material, increased microgliosis, and increased astrogliosis all being particularly apparent in the thalamus at 12 months of age. We previously showed that these neuropathological changes are not present when progranulin levels are knocked down in neuronal cells [19], and the present data now show that selective depletion of progranulin in microglia is also not sufficient to recapitulate this phenotype.

### Changes in gene expression in the brains of aged GrnKO mice are not present in Lyz-cKO mice

**Fig. 5:**
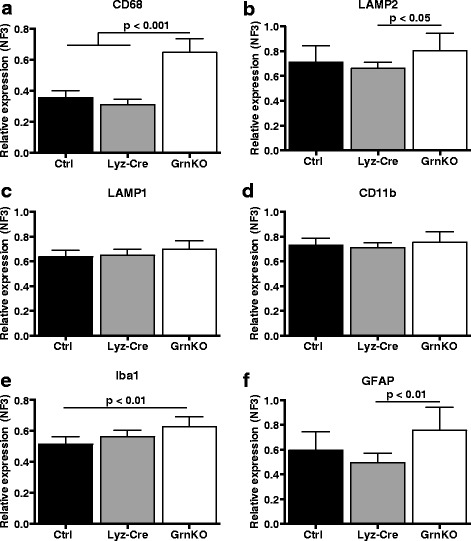
Gene expression changes present in aged GrnKO mice are not recapitulated in Lyz-cKO mice. Evaluation of mRNA expression levels assessed by qRT-PCR of **a** CD68, **b** Lamp2, **c** Lamp1, **d** CD11b, **e** Iba1, and **f** GFAP in the thalamus of 18-month-old mice. *N* = 6 Ctrl, 8 Lyz-cKO, and 8 GrnKO mice. Data represent mean ± SEM. *p* values were calculated using Tukey’s multiple comparison test after one-way ANOVA

Because the depletion of progranulin in the brain in Lyz-cKO animals was not complete, we sought to evaluate additional phenotypic changes related to microglial function using a more sensitive method in older mice. To this end, we evaluated the expression of a panel of six cell-type specific markers and lysosomal proteins in the thalamus of 18-month-old mice by quantitative RT-PCR. We observed significantly increased expression of CD68, a lysosomal protein used as a marker of activated microglia in the brain, in GrnKO mice compared to Ctrl mice, but not in Lyz-cKO mice (Fig. 5a). Lysosomal-associated membrane proteins 1 and 2 (Lamp1 and Lamp2) are lysosomal proteins which are both robustly expressed in the brain [21]. Lamp2 expression was increased in GrnKO but not in Lyz-cKO mice, while Lamp1 expression was not significantly different among the three genotypes (Fig. 5b, c). Evaluation of CD11b expression, an integral membrane protein robustly expressed by microglia, showed no differences between Ctrl, Lyz-cKO, and GrnKO mice (Fig. 5d), but Iba1 expression was significantly increased in GrnKO mice, but not Lyz-cKO mice, compared to Ctrl mice (Fig. 5e). GFAP mRNA expression was more variable than other transcripts measured, though we did observe an increase in GrnKO mice which was statistically significant compared to Lyz-cKO, though not Ctrl, mice (Fig. 5f).

Overall, our results demonstrate increased microgliosis in the thalamus in GrnKO mice that is not re-capitulated in Lyz-cKO mice. Increased expression of Iba1 mRNA was observed in Lyz-cKO brains, but given that other microglia markers did not show the same trend, this may be an effect of Cre expression in microglia and not specific to progranulin knockdown.

### Selective depletion of progranulin in microglia does not recapitulate the hyper-inflammatory phenotype observed in microglia isolated from GrnKO mice

**Fig. 6:**
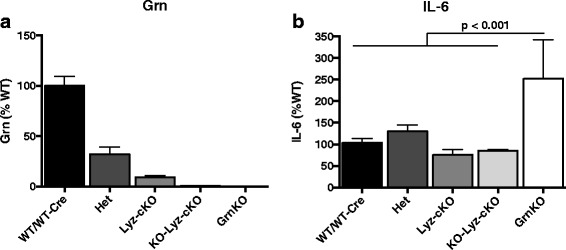
Isolated primary microglia from GrnKO mice display a hyper-inflammatory phenotype that is not present in Lyz-cKO mice*.* **a** Measurement of Grn by ELISA in the conditioned media from primary microglia cultures. **b** Measurement of the inflammatory cytokine IL-6 in the conditioned media of primary microglia cultures after 24 h of stimulation with controlled standard endotoxin (CSE). *N* = 4–9 wells per genotype derived from two independent experiments. Genotypes are defined as follows: WT: Grn^+/+^, Lyz^+/+^; WT-Cre: Grn^+/+^, Lyz^cre/cre^; Het: Grn^+/−^; Lyz-cKO: Grn^flox/flox^, Lyz^cre/cre^; KO-Lyz-cKO: Grn^flox/−^, Lyz^cre/cre^; GrnKO: Grn^−/−^. Data represent mean ± SEM. *p* values were calculated using Tukey’s multiple comparison test after one-way ANOVA

Results from both our previous study examining neuronal-specific knockout mice [19] and from the current study suggest that progranulin acts non-cell autonomously in the brain, that is, that progranulin produced in a single cell type can suppress progranulin-dependent phenotypes in other cell types. However, in Lyz-cKO microglia, progranulin knockdown was incomplete, leaving open the possibility that the residual progranulin expression is sufficient to maintain normal microglia function. We therefore sought to (a) create a mouse model where progranulin expression in microglia was nearly absent and then (b) evaluate isolated primary microglia for a progranulin-dependent phenotype. To this end, we crossed Lyz-cKO mice to GrnKO mice to produce Grn^flox/KO^; Lyz^+/cre^ mice (hereon referred to as KO-Lyz-cKO mice). Primary microglia were cultured from early post-natal WT and WT-Cre (Ctrl), Het, Lyz-cKO, KO-Lyz-cKO, and GrnKO mice. Progranulin secreted from cultured microglia after 24 h was easily detectable by ELISA (Fig. 6a) and expectedly absent in GrnKO cultures. In Lyz-cKO cultures, progranulin expression was reduced to less than 10% that of Ctrl microglia (Fig. 6a), while in KO-Lyz-cKO cultures, progranulin levels were not detectable above background (Fig. 6a). Progranulin mRNA, intracellular protein, and secreted protein levels correlate with each other and accurately reflect gene dosage in microglia derived from heterozygous Grn-null mice (see Additional file 1: Figure S1), providing indirect evidence that recombination of the floxed Grn allele was near complete in this experiment. We then stimulated primary microglia cultures with controlled standard endotoxin (CSE) and measured IL-6 secretion into the media 24 h later. In unstimulated cultures from all genotypes, IL-6 was undetectable in the conditioned media (data not shown). After CSE stimulation, IL-6 was reliably detected by ELISA in conditioned media. GrnKO primary microglia secrete significantly more IL-6 than Ctrl cultures (Fig. 6b). Primary microglia generated from Het, Lyz-cKO, and KO-Lyz-cKO mice secreted similar levels of IL-6 as Ctrl microglia after CSE stimulation (Fig. 6b). Thus, despite near complete knockdown of progranulin expression, primary microglia derived from Lyz-cKO or KO-Lyz-cKO mice do not recapitulate the hyper-inflammatory phenotype observed in GrnKO microglia.

## Discussion

In the present study, we show that reduced progranulin expression in microglia is not sufficient to recapitulate the NCL-like neuropathological features, nor the gene expression changes present in the brains of Grn-null mice. Furthermore, we show that the acute hyper-inflammatory phenotype of isolated Grn-null primary microglia is not recapitulated in progranulin-deficient microglia isolated from myeloid-specific knockout mice, despite near complete ablation of progranulin expression in cultured microglia. These results, combined with our previously published work showing that neuronal-specific knockdown of progranulin is also not sufficient to reproduce the neuropathological features of Grn-null animals, strongly support a non-cell autonomous role for progranulin in the brain.

Some cell-autonomous functions for progranulin have recently been reported. Deficits in social dominance are recapitulated in neuronal-specific Grn-knockout mice [16], while increased self-grooming is recapitulated in microglia-specific Grn-knockout mice [18], suggesting that each of these specific behaviors is dependent on progranulin expression in the given cell type. For the robust neuropathological phenotypes that characterize aged Grn-null mice, it is clear that reduced progranulin expression in neurons or in microglia is not sufficient to recapitulate the observed changes.

Lipofuscin or NCL-like storage material accumulation occurs primarily in post-mitotic neurons and is the result of lysosomal dysfunction [22]. Since neuronal-specific Grn-knockout mice do not display exaggerated lipofuscinosis [16, 19], it appears that extracellularly derived progranulin, presumably from microglia, acts to replace neuron-derived progranulin and maintain lysosomal function. Conversely, since microglia-specific knockdown of progranulin has no effect on lipofuscinosis, it seems that neuron-derived progranulin is also sufficient to maintain normal lysosomal function. The possibility remains that an alternative cell type, either central or peripheral, is responsible for the phenotype of Grn-null mice or for producing progranulin in the brain when expression is knocked down in neurons or microglia. To test this hypothesis, a mouse model with specific deletion of progranulin in both neurons and microglia might be warranted. In addition, we cannot exclude the possibility that other proteins are able to compensate for the loss of progranulin in our model system.

It remains unclear whether progranulin, similar to prosaposin [23], can be derived via sorting at the trans-Golgi network (biosynthetic pathway) as well as from the extracellular space (endocytic pathway) in order to maintain lysosomal function, or whether it acts strictly via the endocytic pathway, independent of the cell type it is derived from. Also unclear is whether progranulin’s role in lysosomal biology is its only role in neurons and whether or not lysosomal dysfunction is a driving pathological force in the development of FTLD. Accumulation of autofluorescent storage material in the tissues of FTLD patients has recently been reported [24, 25], but a direct connection between storage material accumulation, lysosomal function, and the pathophysiology of FTLD is not yet established. Lipofuscin accumulation may occur with partial loss of progranulin expression [16, 24, 25], but the dramatic increase in accumulation in Grn-null mice and the dramatically different clinical presentation of *GRN*-null patients with NCL compared to heterozygous *GRN*-null patients with FTLD do not support a direct dose dependence of these phenotypes on progranulin expression levels. The surprising observation that progranulin expression is actually increased in affected brain regions in FLTD patients with *GRN* mutations due to increased expression from activated microglia [26] strongly supports the hypothesis that partial loss of *GRN* expression in neurons plays a cell-intrinsic role critical to sustained neuronal health.

It is important to note that progranulin depletion in cells of myeloid lineage, which include microglia, was incomplete in this study, and thus, there remains the possibility that more complete cell type-specific knockdown of progranulin could lead to neuropathological changes in the brain. The LyzM promotor driving Cre recombinase expression produces incomplete recombination in microglia [27], similar to the level of recombination we observed in this study (Fig. 1). Future studies might further evaluate the effect of reducing progranulin expression in microglia using a promotor such as Cx3cr1 driving Cre expression for more complete knockdown [27]. The Lyz-cKO mice used in this study still displayed reduced progranulin expression in microglia, and our data clearly show that this intervention is insufficient to recapitulate the NCL-like neuropathology that is characteristic of Grn-null mice.

We achieved much more complete knockdown of progranulin in isolated primary microglia cultures, in particular from KO-Lyz-cKO mice, and even in this instance, did not observe the same phenotype that is observed in Grn-null microglia. The lack of a hyper-inflammatory phenotype in isolated microglia with selective depletion of Grn, despite a robust phenotype in constitutive Grn-null microglia, cannot be attributed to the presence of secreted progranulin derived from other cell types as no other cell types are present in this system. Instead, it may be that microglia that develop in a progranulin-rich environment have a normal response to an inflammatory stimulus. It has been reported that AAV-mediated over-expression of progranulin in Grn-null microglia reduced cytokine secretion after inflammatory stimulation [20], indicating a specific role for intrinsic progranulin expression in microglia in modulating cytokine expression. Still, there may be a critical period in microglia development when exposure to circulating progranulin plays a role in shaping part of the inflammatory response mechanisms, and that after this critical period, exposure to progranulin is dispensable for normal cytokine expression and release. This hypothesis has not yet been tested but remains a potential area of future investigation.

## Conclusion

We have shown that knockdown of progranulin in myeloid-lineage cells including microglia is not sufficient to recapitulate the neuropathological abnormalities, gene expression changes, or the hyper-inflammatory profile of isolated microglia that are present in constitutive Grn-null mice. This data, when combined with our previous work and that of others, suggests that progranulin acts non-cell autonomously in the brain in some instances and adds a layer of complexity to understanding progranulin-dependent phenotypes in the brain.

## Methods

### Mice

The generation of “floxed” progranulin-targeted (Floxed) mice was previously described [28]. Mice expressing Cre recombinase knocked in to the Lys2 locus (referred to as the LysMcre allele) were obtained from The Jackson Laboratory (B6.129P2-*Lyz2* ^*tm1(cre)Ifo*^/J). Homozygous Grn^flox/flox^ mice were crossed to Lyz2^+/cre^ mice, and resultant pups heterozygous at both loci were then intercrossed to produce Grn^flox/flox^; Lyz2^cre/cre^ mice. For some experiments, homozygous Grn^flox/flox^; Lyz2^cre/cre^ mice were crossed to Grn^−/−^ animals [28], and resultant offspring heterozygous at all three loci were intercrossed to produce littermates of mixed genotypes. Final experimental cohorts were comprised of mice from homozygous matings of Grn^flox/flox^; Lyz2^cre/cre^ mice (referred to as Lyz-cKO); heterozygous crosses of Grn^flox/−^; Lyz2^+/cre^ x Grn^flox/−^; Lyz2^+/cre^ mice, from which Lyz-cKO, Floxed, and Grn^−/−^ with or without the Cre transgene (referred to as GrnKO) were selected as experimental animals; and Grn^+/−^ x Grn^+/−^ crosses.

As we have previously shown that Grn^+/+^ (WT) and Grn^flox/flox^ (Floxed) mice are not significantly different in progranulin expression levels or neuropathology [19], WT and Floxed mice were grouped together and referred to as control (Ctrl) mice. Grn^+/−^ (Het) mice were included for reference when evaluating progranulin levels. For experiments using primary microglia cultures, mice homozygous for the LysMCre allele but wild-type at the Grn locus (WT-Cre) were used as controls to exclude the possibility that expression of Cre in microglia alters cytokine release. Genotyping was performed on tail tip DNA at wean and confirmed on a second DNA sample at sacrifice using primer sequences given in Table 1.

**Table 1:** Primer sequences for genotyping and quantitative RT-PCR

Genotyping		
Gene	Forward primer sequence	Reverse primer sequence
Grn	Common: 5’-CGGAACACAGTGTCCAGATG-3’	Intron 2: 5’-ATCAACCAAAGGGTCTGTGC-3’ Exon 5: 5’-GTGGCAGAGTCAGGACATTCAAACT-3’
Lys2	WT: 5’-TTACAGTCGGCCAGGCTGAC-3’ Cre: 5’-CCCAGAAATGCCAGATTACG-3’	Common: 5’- CTTGGGCTGCCAGAATTTCTC-3’
Quantitative RT-PCR		
Gene	Forward primer sequence	Reverse primer sequence
CD11b	5’-ATCCCCCTGCAAGACAGTGA-3’	5’-AGCAGTCAGGCAGGGACATG-3’
CD68	5’-GTGCTCATCGCCTTCTGCATCA-3’	5’-GGCGCTCCTTGGTGGCTTAC-3’
Gfap	5’-GAGTGGTATCGGTCTAAGTTTGCA-3’	5’-CGATAGTCGTTAGCTTCGTGCTT-3’
Grn	5’-CTGTAGTGCAGATGGGAAATCCTGCT-3’	5’-GTGGCAGAGTCAGGACATTCAAACT-3’
Iba1	5’-GTCCTTGAAGCGAATGCTGG-3’	5’-CATTCTCAAGATGGCAGATC-3’
Lamp1	5’-ACATCAGCCCAAATGACACA-3’	5’-GGCTAGAGCTGGCATTCATC-3’
Lamp2	5’-AGCACAGTATTTCCTGGTGCT-3’	5’-CGACAGGAGTCAGGTTGTAAGTTAA-3’
RplI13a	5’-GGAGGAGAAACGGAAGGAAAAG-3’	5’-CCGTAACCTCAAGATCTCGTTCTT-3’
Usf1	5’-CCTGTGGCGTGGCAGTCT-3’	5’-TGCACGCCCACACTGTTT-3’
Zfp91	5’-TCCTGTGGGCGACTCTTCAG-3’	5’-TAATCCCTCTGGTCTGTATGATGCT-3’

Mice were housed on ventilated racks in specific pathogen-free barrier facility with a 12-h light/dark cycle. Mice were group-housed with their littermates to a maximum of four mice per cage.

### Isolation of adult murine microglia

Mice were sacrificed by CO_2_ inhalation followed by cervical dislocation. Whole brains were removed and briefly washed in 1 mL of Hank’s buffered saline solution (HBSS) before being placed in Liberase (0.1 M HBSS, 47.7 μL/mL reconstituted Liberase). After being rotated for 45 min at 37 °C, brains were mechanically homogenized using a P1000 pipette tip until tissue was dissociated, then spun at 200g for 5 min at 18 °C. Supernatant was removed, and homogenates were re-suspended in 2 mL of re-suspension buffer (0.1 M HBSS, 0.5 μL/mL filtered MgCl_2_) and filtered through a 70 μm cell strainer. The sample tube and cell strainer were both washed with additional re-suspension buffer, after which the combined homogenates were spun at 200g for 5 min at 18 °C.

Following homogenization, the supernatant was removed, samples were re-suspended in FACS buffer (0.1 M PBS, 1 mM EDTA, 1% BSA), 500 μL of Miltenyi® Myelin Removal Beads II were added to the solution, and samples were incubated at 4 °C for 15 min. Samples were then washed with FACS buffer and spun at 200g for 10 min at 18 °C. Supernatants were removed and pellets were re-suspended in FACS buffer prior to myelin depletion using the AutoMACS (Miltenyi; Deplete_S program). Following the automated magnetic separation, the negative fraction was collected and spun at 200g for 5 min at 18 °C.

Following myelin removal, samples were incubated with 30 μL of Miltenyi® CD11b magnetic beads in 270 μL of FACS buffer for 15 min at 4 °C, after which they were washed with 2 mL of FACS buffer and spun at 200g for 10 min at 18 °C. Cells were re-suspended in 500 μL of FACS buffer prior to AutoMACS selection using the Possel_S program, after which the CD11b-positive portion was collected. The samples were spun at 200g for 5 min at 18 °C and re-suspended in 100 μL of FACS buffer for subsequent antibody staining.

The cells were incubated with Ebioscience® CD11b-PE and Ebioscience® CD45-APC antibodies at a dilution of 1:1000 for 15 min at 4 °C. An additional 150 μL of FACS buffer was added along with Ebioscience® 7AAD Viability Dye at a dilution of 1:250. The sample was then subjected to flow cytometry sorting using a FACSAria machine, with an 85 nozzle and 45 psi setting, with CD45^low^ CD11b^+^ cells isolated as the population of interest.

### Protein extraction and quantification of Grn by ELISA

Whole brain lysate was prepared from ten WT and/or Floxed mice, six Lyz-cKO mice, and four GrnKO mice at 3–4 months of age by homogenizing previously snap-frozen brains in a rotor-stator homogenizer for 30 s in 1 mL of complete lysis buffer (50 mM Tris-HCl, 1% Triton-X, 150 mM NaCl, Halt phosphatase inhibitor cocktail (Thermo Fisher Scientific), Halt protease inhibitor cocktail (Thermo Fisher Scientific)). Total protein was assayed using Bradford reagent (BioRad).

Microglia isolated by flow cytometry from four WT, four Lyz-cKO, and four Het mice at 3–4 months of age were pooled and lysed in 100 μL of complete lysis buffer, then stored at − 80 °C until used.

The quantity of Grn in whole brain lysate, sorted cell lysate, or conditioned media was determined by an enzyme-linked immunosorbent assay (ELISA) using a commercially available kit (Mouse progranulin ELISA; Adipogen, Korea). Microglia supernatant samples were diluted 1:5; for whole brain lysate, 100 μg of protein was used; for cell lysates from sorted microglia, the entire sample minus a small aliquot for protein quantification was loaded and results normalized to total protein, typically 5–10μg. All samples were run in duplicate. The ELISA was conducted according to the manufacturer’s instructions. Data represent the average per condition, and all conditions that were compared directly were run on the same plate.

### RNA isolation and qPCR

For analysis of *Grn* mRNA expression, whole brain tissue (four Floxed, three Lyz-cKO, and two GrnKO mice) or microglia isolated from adult brain (four WT, four Lyz-cKO, four GrnKO) from mice 3–4 months of age were collected; for analysis of cell-type specific and lysosomal transcripts, the thalamus was micro-dissected from six WT and/or Floxed, eight Lyz-cKO, and eight GrnKO mice at 18 months of age. Tissue samples were immediately frozen at − 80 °C. Samples were homogenized with a bead homogenizer in lysis buffer followed by total RNA extraction (PureLink RNA mini kit; Invitrogen) performed according to the manufacturer’s instructions. Reverse transcription of all samples was carried out using the Superscript VILO kit (Invitrogen) according to the manufacturer’s instructions, using 1 μg of total RNA as input for cDNA synthesis. Following this, cDNA was diluted 1:10 in ddH_2_O for a total input of 5 ng into the quantitative PCR reaction, done using FastSybr (Applied Biosystems), and conducted on a Step-One ABI System (Applied Biosystems). Quantification of mRNA levels was accomplished using the standard curve method, with amplification of target mRNA and control genes in separate wells. Each sample was run in duplicate. The relative amount of mRNA in each well was calculated as the ratio between target mRNA and a normalization factor created using three control genes (*Usf1*, *RplI13a*, and *Zfp91*) based on GeNorm [29]. Values are presented as % WT and/or Floxed control. All primer sequences are provided in Table 1.

### Immunohistochemistry

Immunohistochemistry was performed as previously described [19]. Briefly, 25 μm floating sections were placed in net-well inserts and washed for 10 min in phosphate-buffered saline (PBS). After quenching, endogenous peroxidase activity was quenched with 3% H_2_O_2_ for 45 min, sections were blocked in 5% normal serum and 5% bovine serum albumin, followed by overnight incubation shaking at room temperature in primary antibody diluted in 5% normal serum. After two 15 min washes, secondary antibody diluted in 1% normal serum and PBS with 0.01% Triton X (PBS-T) was applied for 2 h shaking at room temperature. Sections were washed for 30 min in PBS before an amplification step was performed using an avidin–biotin–horseradish peroxidase complex kit (Vector Laboratories). Colorimetric detection was achieved with the peroxidase substrate kit Vector DAB (Vector Laboratories) according to the manufacturer’s instructions. Sections were mounted by hand on onto glass slides (Fisherbrand Superfrost Plus) and dried overnight before being dehydrated through a series of alcohols and xylene, and cover-slipped with DEPEX (Electron Microscopy Sciences). Antibodies used were as follows: the microglia marker Iba1 (Wako; 1:2000, rabbit polyclonal), the astrocyte marker GFAP (Sigma; 1:2000, mouse monoclonal), and appropriate biotinylated secondary antibodies (Vector, 1:2000).

Sections to be assessed for autofluorescence were mounted onto glass slides, washed in PBS-T for 30 min, and then stained with DAPI in PBS at 1:10,000 for 5 min. Slides were washed in twice in PBS for 5 min prior to coverslipping.

### Image acquisition and analysis

Images were acquired as previously described [12]. Integrated optical density measurements of signal intensity were acquired as previously described [8] to quantify autofluorescence, a surrogate for lipofuscin deposition, in 4 images per mouse taken from 5 WT/Floxed, 12 Lyz-cKO, and 4 GrnKO mice. For quantification of colorimetric stains (Iba1 and GFAP), a threshold was set that pseudo-colored stained areas within a defined region of interest in each image. The average percent thresholded area for 4–6 images per mouse taken from 5 WT/Floxed, 12 Lyz-cKO, and 4 GrnKO mice is reported.

### Primary microglia isolation and stimulation

Primary microglia cultures were generated from postnatal day 0–3 pups as previously described [30]. Cells were stimulated with controlled standard endotoxin (final concentration 100 ng/ml; Associates of Cape Cod, MA, USA) as previously described [30], and conditioned media was collected after 24 h and stored at − 20 °C until used. The quantity of IL-6 in the conditioned media was measured using a commercially available ELISA (Ready-set-go IL6 ELISA, eBiosciences, San Diego, USA) according to the manufacturer’s instructions. Results are presented from two independent experiments with 3–6 wells per genotype in each experiment.

### Statistical analysis

All statistical comparisons were performed as a one-way analysis of variance (ANOVA) with Tukey post-hoc analysis to compare individual means to control and correct for multiple comparisons (Prism 6, Graphpad Software Inc.). A *p* value less than 0.05 was considered significant.

## Additional file

Additional file 1: Figure S1. Progranulin mRNA, intracellular, and secreted protein levels correlate in primary microglia cultures. (A) Progranulin mRNA levels were reduced by approximately 50% in Het cultures compared to WT cultures and not detectable in GrnKO cultures. *N* = 2–3 wells/per genotype. (B) Progranulin protein quantified by ELISA on cell lysate and normalized to total protein per well shows a corresponding decrease of about 50% in Het cultures compared to WT cultures and negligible levels in GrnKO cultures. *N* = 6 wells/genotype. (C) Secreted progranulin detected by ELISA on conditioned media again shows progranulin reduced to approximately 50% in Het cultures compared to WT cultures and not detectable in GrnKO cultures. *N* = 6 wells/genotype*.* (PDF 90 kb)
