# Supplementary figures and images for: Selective depletion of microglial progranulin in mice is not sufficient to cause neuronal ceroid lipofuscinosis or neuroinflammation
Source: J Neuroinflammation. 2017 Nov 17;14:225. doi: 10.1186/s12974-017-1000-9 (PMC5693502; doi:10.1186/s12974-017-1000-9)

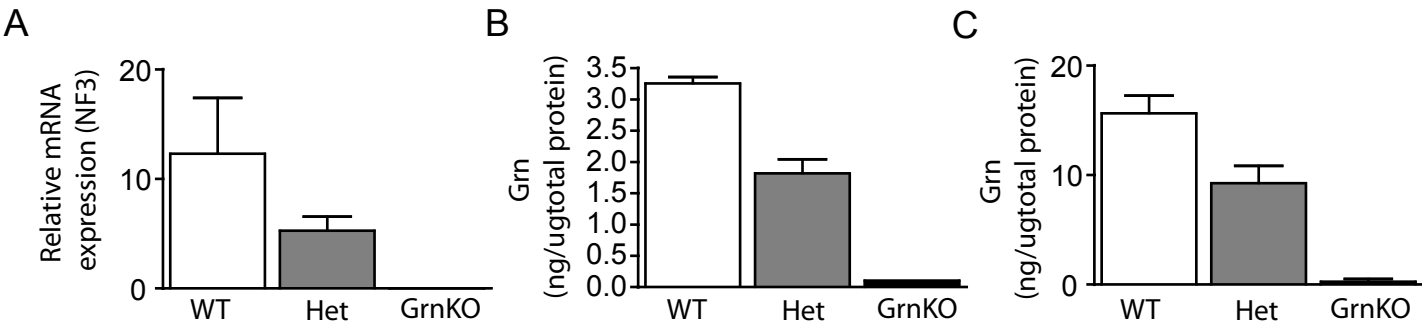

Supplement: Additional file 1: Figure S1. — Progranulin mRNA, intracellular, and secreted protein levels correlate in primary microglia cultures. (A) Progranulin mRNA levels were reduced by approximately 50% in Het cultures compared to WT cultures and not detectable in GrnKO cultures. N = 2–3 wells/per genotype. (B) Progranulin protein quantified by ELISA on cell lysate and normalized to total protein per well shows a corresponding decrease of about 50% in Het cultures compared to WT cultures and negligible levels in GrnKO cultures. N = 6 wells/genotype. (C) Secreted progranulin detected by ELISA on conditioned media again shows progranulin reduced to approximately 50% in Het cultures compared to WT cultures and not detectable in GrnKO cultures. N = 6 wells/genotype. (PDF 90 kb) [file 12974_2017_1000_MOESM1_ESM.pdf]
